# An Update on the Adult-Onset Hereditary Cerebellar Ataxias: Novel Genetic Causes and New Diagnostic Approaches

**DOI:** 10.1007/s12311-024-01703-z

**Published:** 2024-05-18

**Authors:** Laura Ivete Rudaks, Dennis Yeow, Karl Ng, Ira W. Deveson, Marina L. Kennerson, Kishore Raj Kumar

**Affiliations:** 1 Molecular Medicine Laboratory and Neurology Department, Concord Repatriation General Hospital, Sydney, Australia; 2 Faculty of Medicine and Health, The University of Sydney, Sydney, Australia; 3 Genomics and Inherited Disease Program, The Garvan Institute of Medical Research, Sydney, Australia; 4 Clinical Genetics Unit, Royal North Shore Hospital, Sydney, Australia; 5 Neurodegenerative Service, Prince of Wales Hospital, Sydney, Australia; 6 Neuroscience Research Australia, Sydney, Australia; 7 Neurology Department, Royal North Shore Hospital, Sydney, Australia; 8 Faculty of Medicine, University of New South Wales, Sydney, Australia; 9 The Northcott Neuroscience Laboratory, ANZAC Research Institute, Sydney Local Health District, Sydney, Australia; 10 Faculty of Medicine, St Vincent’s Healthcare Campus, UNSW Sydney, Sydney, Australia

**Keywords:** Ataxia, Cerebellar ataxia, Spinocerebellar ataxia type 4, Spinocerebellar ataxia type 27B, *RFC1*, *THAP11*

## Abstract

The hereditary cerebellar ataxias (HCAs) are rare, progressive neurologic disorders caused by variants in many different genes. Inheritance may follow autosomal dominant, autosomal recessive, X-linked or mitochondrial patterns. The list of genes associated with adult-onset cerebellar ataxia is continuously growing, with several new genes discovered in the last few years. This includes short-tandem repeat (STR) expansions in *RFC1*, causing cerebellar ataxia, neuropathy, vestibular areflexia syndrome (CANVAS), *FGF14*-GAA causing spinocerebellar ataxia type 27B (SCA27B), and *THAP11*. In addition, the genetic basis for SCA4, has recently been identified as a STR expansion in *ZFHX3.* Given the large and growing number of genes, and different gene variant types, the approach to diagnostic testing for adult-onset HCA can be complex. Testing methods include targeted evaluation of STR expansions (e.g. SCAs, Friedreich ataxia, fragile X-associated tremor/ataxia syndrome, dentatorubral-pallidoluysian atrophy), next generation sequencing for conventional variants, which may include targeted gene panels, whole exome, or whole genome sequencing, followed by various potential additional tests. This review proposes a diagnostic approach for clinical testing, highlights the challenges with current testing technologies, and discusses future advances which may overcome these limitations. Implementing long-read sequencing has the potential to transform the diagnostic approach in HCA, with the overall aim to improve the diagnostic yield.

## Introduction

The adult-onset hereditary cerebellar ataxias (HCAs) are rare, progressive neurologic disorders which can be inherited in an autosomal dominant (AD), autosomal recessive (AR), X-linked or mitochondrial pattern. The prevalence of AD-HCA has been reported as up to 5.6/100,000 and AR-HCA as up to 7.2/100,000 in various studies, with an estimated global average of 2.7/100,000 for AD-HCA and 3.3/100,000 for AR-HCA [1–6]. However, the reported prevalence may vary between studies due to different inclusion criteria, as well as variability in HCA prevalence in different geographic regions or ethnic groups [1, 4–6]. There is an extensive list of potential causative genes, and this list continues to grow, with several new ataxia genes recently identified using advanced genomic technologies [7–11]. With improvements in knowledge of ataxia genes, and developments in genetic testing technologies within the last couple of decades, further updates in epidemiological estimates will be required to accurately determine the global disease burden of HCA [4–6].

There is significant overlap in clinical features between different genetic forms of HCA and phenotypic heterogeneity within genetic forms. Therefore, reliable prediction of the causative gene is usually not feasible from the clinical evaluation alone and screening of a broad range of genes is usually undertaken, except in cases with a known genetic diagnosis in the family. The diagnostic yield of genetic testing amongst individuals with suspected HCA is highly variable, ranging from 28.8–70.5% [12–16]. The diagnostic process can be complex, with several tests required to ensure comprehensive evaluation for the various genetic variant types, including short tandem repeat (STR) expansions, single nucleotide variants, insertions, deletions, and duplications. However, despite thorough evaluation using traditional approaches, there remains a significant diagnostic gap.

In this review, we provide an overview of the genetic basis of adult-onset HCAs. We discuss genetic forms of ataxia related to recently discovered genes or gene variant types, including STR expansions in *RFC1* (CANVAS), *FGF14* (SCA27B) and *THAP11* (‘SCA51’), as well as discussing SCA4, for which the genetic basis has only recently been discovered [10]. We propose a diagnostic approach for individuals presenting with adult-onset HCA, discuss the role of advanced genomic technologies, including long-read sequencing, and outline future directions in the diagnosis of adult-onset ataxias.

## Autosomal Dominant Spinocerebellar Ataxias (SCAs)

The AD HCAs, also termed the spinocerebellar ataxias (SCAs), are numbered in order of their discovery, with the latest being SCA50 (OMIM phenotypic series PS164400) (Table 1). The most common SCAs are caused by (CAG)_n_ STR expansions within coding regions of various genes, with SCA3 the most common form globally, accounting for 20–50% of AD ataxia [1, 17]. The (CAG)_n_ STR expansion results in a polyglutamine (polyQ) stretch within the protein product, and these are referred to as ‘polyglutamine SCAs’ [18]. Several other SCAs are caused by STR expansions in non-coding regions, including untranslated or intronic regions (Table 1)[19]. Numerous SCAs are also caused by conventional variants (i.e. non-STR expansions), including single nucleotide variants, insertions, deletions, and duplications, although these are estimated to account for less than 6% of SCAs [17, 19, 20].

**Table 1:** Autosomal dominant spinocerebellar ataxias

Phenotype	Gene	Variant type	Locus	Phenotype MIM
SCA1	*ATXN1*	(CAG)_n_ repeat	6p22.3	164400
SCA2	*ATXN2*	(CAG)_n_ repeat	12q24.12	183090
SCA3 (Machado-Joseph disease)	*ATXN3*	(CAG)_n_ repeat	14q32.12	109150
SCA4	*ZFHX3*	(GGC)_n_ repeat	16q22.2-q22.3	600223
SCA5	*SPTBN2*	Missense, deletion	11q13.2	600224
SCA6	*CACNA1A*	(CAG)_n_ repeat *Allelic disorders EA2 and FHM occur with point mutations/ deletions* [63, 64]	19p13.13	183086
SCA7	*ATXN7*	(CAG)_n_ repeat	3p14.1	164500
SCA8	*ATXN8/ ATXN8OS*	(CAG)_n_/(CTG)_n_ repeat	13q21/ 13q21.33	608768
SCA9	Unknown	Unknown	Not mapped	612876
SCA10	*ATXN10*	Non-coding (ATTCT)_n_ repeat	22q13.31	603516
SCA11	*TTBK2*	Insertion, deletion	15q15.2	604432
SCA12	*PPP2R2B*	Non-coding (CAG)_n_ repeat	5q32	604326
SCA13	*KCNC3*	Missense	19q13.33	605259
SCA14	*PRKCG*	Missense, deletion	19q13.42	605361
SCA15/16	*ITPR1*	Missense, deletion *Allelic disorder SCA29 caused by missense variants*	3p26.1	606658
SCA17	*TBP*	(CAG)_n_ repeat	6q27	607136
SCA18	Unknown – *IFRD1* candidate gene	Missense variant in candidate gene *IFRD1* identified in one family [109]	7q22-q32	607458
SCA19/22	*KCND3*	Missense, deletion	1p13.2	607346
SCA20	12 genes	260-kb duplication identified in one reported family [110]	11q12	608687
SCA21	*TMEM240*	Missense, nonsense	1p36.33	607454
SCA23	*PDYN*	Missense	20p13	610245
SCA24 – reassigned SCAR4	*-*	-	-	-
SCA25	*PNPT1*	Splice site	2p16.1	608703
SCA26	*EEF2*	Missense	19p13.3	609306
SCA27A	*FGF14*	Missense, nonsense, insertion, deletion	13q33.1	193003
SCA27B	*FGF14*	Non-coding (GAA)_n_ repeat	13q33.1	620174
SCA28	*AFG3L2*	Missense, duplication	18p11.21	610246
SCA29	*ITPR1*	Missense *Allelic disorder SCA15 caused by missense variants or deletions*	3p26.1	117360
SCA30	Unknown		4q34.3-q35.1	613371
SCA31	*BEAN1*	Non-coding (TGGAA)_n_ repeat	16q21	117210
SCA32	Unknown	-	7q32-q33	613909
SCA33* – not assigned	*-*	-	-	-
SCA34	*ELOVL4*	Missense	6q14.1	133190
SCA35	*TGM6*	Missense, deletion	20p13	613908
SCA36	*NOP56*	Non-coding (GGCCTG)_n_ repeat	20p13	614153
SCA37	*DAB1*	Non-coding (ATTTC)_n_ repeat	1p32.2-p32.1	615945
SCA38	*ELOVL5*	Missense	6p12.1	615957
SCA39*	44 genes	7.5 Mb duplication identified in one family [111]	11q21-q22.3	-
SCA40	*CCDC88C*	Missense	14q32.11-q32.12	616053
SCA41	*TRPC3*	Missense	4q27	616410
SCA42	*CACNA1G*	Missense	17q21.33	616795
SCA43	*MME*	Missense	3q25.2	617018
SCA44	*GRMR1*	Missense, duplication	6q24.3	617691
SCA45	*FAT2*	Missense	5q33.1	617769
SCA46	*PLD3*	Missense	19q13.2	617770
SCA47	*PUM1*	Missense	1p35.2	617931
SCA48	*STUB1*	Missense, nonsense, insertion, deletion, duplication	16p13.3	618093
SCA49	*SAMD9L*	Missense	7q21.2	619806
SCA50	*NPTX1*	Missense	17q25.3	620158
SCA51*	*THAP11*	(CAG)_n_ repeat	16q22.1	-
DRPLA	*ATN1*	(CAG)_n_ repeat	12p13.31	125370
**Other autosomal dominant complex ataxias**				
ADCADN	*DNMT1*	Missense	19p13.2	604121
ATXPC	*SAMD9L*	Missense	7q21.2	159550
SPAX-1	*VAMP1*	Splice site	12p13.31	108600

ADCADN: autosomal dominant cerebellar ataxia, deafness, and narcolepsy; ATXPC: ataxia-pancytopenia syndrome; DRPLA: dentatorubral-pallidoluysian atrophy; EA2: episodic ataxia type 2; FHM: familial hemiplegic migraine; SCAR4: autosomal recessive spinocerebellar ataxia-4; SPAX-1: autosomal dominant spastic ataxia-1

^*^Not yet assigned on Online Mendelian Inheritance in Man® (OMIM®)

Although there are a large number of different genes, the causative pathways converge on the common outcome of cerebellar Purkinje cell loss, accounting for the core clinical features seen in the SCAs [19]. The SCAs generally present with progressive cerebellar ataxia, manifesting as gait ataxia, impaired limb coordination, dysarthria, and cerebellar ocular findings with cerebellar atrophy on cranial imaging [18, 20]. Certain genetic subtypes are associated with a pure form of cerebellar ataxia, whilst others are associated with additional neurological and non-neurological features, including pyramidal tract signs, peripheral neuropathy, movement disorders/parkinsonism, cognitive impairment, dysautonomia and seizures [21]. The onset of polyQ SCAs is typically in the third or fourth decade but can also occur in childhood or later adulthood, with longer repeat lengths associated with earlier disease onset and faster symptom progression [17, 18, 20]. The polyQ SCAs are also associated with the phenomenon of anticipation, in which disease onset becomes earlier, or severity increases, in subsequent generations [20]. The non-polyQ SCAs are associated with greater phenotypic heterogeneity, for example, associated non-ataxia features, such as cognitive impairment, demonstrate marked variability in expression for patients with the same affected gene [22]. Although age at onset can have a broad range, compared to polyQ SCAs, the non-polyQ SCAs generally have an earlier age of onset with slower progression and are less severe [18, 20, 22]. They are generally not associated with anticipation, although there are exceptions such as SCA10 and SCA37, which are caused by pentanucleotide STR expansions [23, 24].

For several STR expansion disorders, the penetrance (i.e. the proportion of individuals with the variant that develop disease manifestations) is incomplete and may vary based on repeat length [25]. SCA17, is caused by (CAG)_n_ STR expansions in *TBP*, with sequences also containing CAA interruptions. Full penetrance is seen in individuals with ≥ 49 CAG/CAA repeats, whist penetrance is incomplete in those with 41–48 repeats (i.e. ‘intermediate’ range), and 50% of individuals remain asymptomatic at age 50 [25, 26]. Furthermore, it has been shown that approximately 1–2% of the population carry a *TBP* STR expansion in the ‘intermediate’ range [25, 27]. Interestingly, the variability in penetrance may be partially explained by a digenic phenomenon, with a concurrent variant in *STUB1* identified in 49–87% of SCA17-manifesting individuals with ‘intermediate’ expansions [25, 28]. Non-repeat variants in *STUB1* have been associated with both AD and AR forms of HCA (SCA48 and ATX-*STUB1*/SCAR16 respectively) [29, 30]. However, the identification of the digenic inheritance (DI) of *TBP*/*STUB1*, or ‘SCA17-DI’, suggests concurrent variants in *STUB1* may result in disease expression in individuals with ‘intermediate’ *TBP* expansions, or alternatively, that *TBP* ‘intermediate’ expansions may be a disease modifier for SCA48 [25, 28]. This discovery has implications for genetic testing. For individuals with ‘intermediate’ range *TBP* expansions, further sequencing of *STUB1* could be undertaken, whilst in individuals with *STUB1* variants, *TBP* STR expansions could be evaluated.

Beyond variability in prevalence, ‘intermediate’ STR expansions may confer risk for other neurologic conditions. *ATXN2* STR expansions of ≥ 33 repeats can cause SCA2, whilst ≥ 31 repeats confer increased risk for amyotrophic lateral sclerosis (ALS), as well as frontotemporal dementia [31–33]. Similarly, SCA1 occurs with *ATXN1* STR expansions of ≥ 39 repeats, whilst an increased risk of ALS has been identified in individuals with ≥ 32 repeats [34, 35]. *ATXN2* STR expansions ≥ 32 repeats have also been associated with parkinsonism, without cerebellar ataxia [20, 36]. Furthermore, in addition to SCA17 with reduced penetrance, ‘intermediate’ *TBP* STR expansions have been suggested as a susceptibility factor for parkinsonism [37].

In the last couple of years, several new SCA subtypes have been discovered. In the following section, we will discuss SCA27B, due to a STR expansion in *FGF14*, and ataxia due to a STR expansion in *THAP11*, proposed as ‘SCA51’ [38]. We also describe the recent discovery of the genetic cause underpinning SCA4.

### SCA27B

Although *FGF14* has a long-established link with ataxia, with conventional variants (point mutations, insertions and deletions) known to cause the rare SCA27A (formerly SCA27), it has been recently discovered that a STR expansion in intron 1 of *FGF14* also results in an AD HCA, SCA27B [39–41]. SCA27B likely reflects one of the most common causes of adult-onset HCA, identified in 13.7–15%, 14.4–18%, 28%, 11.9%, 10%, 9%, 0–1.2% and 61% of Australian, German, Spanish, Greek, Indian, Brazilian, Japanese and French-Canadian adult-onset ataxia cohorts respectively, with the higher French-Canadian prevalence suspected related to a founder effect [8, 41–44]. It is caused by a (GAA)_n_ intronic STR expansion in *FGF14*, with repeat lengths > 250 identified as pathogenic, albeit with reduced penetrance, and a fully-penetrant pathogenic threshold of > 300 repeats [8, 41, 42]. The lower limits of this pathogenic threshold remain to be confirmed, with recent reports identifying symptomatic individuals within the 200–249 repeat range [45, 46]. Intergenerational transmission tends to result in further STR expansion in the case of maternal transmission, and contraction upon paternal transmission [42]. In comparison, non-GAA repeats or “interrupted GAA repeat expansions”, such as (GAAGGA)n and [(GAA)n(GCA)m]z, did not segregate with disease and therefore may not be pathogenic [47–49].

In SCA27B, onset of progressive ataxia occurs at a mean age of 60 years (range 30–88) [8, 50]. Several studies have suggested that repeat length is inversely correlated to age at onset [8, 41, 51]. Clinical features include gait ataxia, limb ataxia, oculomotor abnormalities, with down-beat nystagmus seen in 42%, gaze-evoked nystagmus in 55%, and dysarthria in approximately 50% [8, 41]. Ataxia may be episodic at onset (13–46%) and precede permanent ataxia by several years [8, 50]. Episodes include combinations of gait and limb ataxia, dysarthria, vertigo, and diplopia with a duration of minutes to days [8]. Lower limb pyramidal signs are identified in some individuals, and vestibular hypofunction is present in 22–60%, but cough is not a common feature (whereas this is often seen in *RFC1*-related CANVAS) [8, 41, 50]. Autonomic dysfunction is rare at onset, or in early stages, but becomes more frequent with disease progression [41, 50]. In the majority, cranial imaging demonstrates cerebellar atrophy, particularly affecting the vermis, but commonly also involving cerebellar hemispheres [8, 50]. Progression is relatively slow, with wheelchair dependence rare, and restricted to those with advanced disease of greater than 10 years [50].

The protein product, FGF14, plays a role in the function of voltage-gated sodium channels and consequent rhythmic spontaneous firing of Purkinje cells [8]. Noting channelopathies underlie the episodic ataxias, it therefore is not surprising that impaired channel function may also result in episodic features in SCA27B [52]. Treatment with 4-aminopyridine (4-AP) has been trialled in numerous individuals with SCA27B, with good clinical response in most patients, particularly with reduced frequency or severity of episodic symptoms [8, 50]. It is hypothesised that 4-AP, which is known to block Kv1 potassium channels, may exert its therapeutic effect through amelioration of firing deficits of Purkinje neurons [50]. Treatment with acetazolamide has also demonstrated benefit in 44% of individuals in one series [53].

### Comparison between SCA27A and SCA27B

Although both SCA27A and SCA27B are caused by variants in *FGF14*, there are notable phenotypic differences (Table 2). SCA27A, tends to occur earlier in life, usually in childhood or early adulthood, although onset up to age 50 has been reported [54, 55]. Similar to SCA27B, individuals may present with episodic ataxia, which occurs in 21% [56]. Indeed, in some individuals, episodic ataxia may dominate the clinical picture, leading previous authors to suggest such cases be categorised as a new EA disorder and considered as a separate entity to the permanent cerebellar ataxia syndrome of SCA27A [55, 57–59]. Although only trialled in few cases, episodes can respond to acetazolamide, but may also settle spontaneously with age [55, 59–61]. Overall in SCA27A, tremor is a prominent feature, present in 96%, and is the presenting feature in 58%, with mean age at onset 12.1 years [56]. Ataxia tends to occur later, with mean onset at 23.7 years [56]. Although horizontal nystagmus is common, down-beat nystagmus has only rarely been reported, and appears to accompany presentations with episodic ataxia [56, 60, 61]. Progression is relatively slow, with less than 14% progressing to severe gait impairment [56]. In summary, the two forms of SCA27 differ in age at onset, with a different, but overlapping spectrum of clinical features.

**Table 2:** Clinical and genetic features of SCA27A and SCA27B

Clinical feature	SCA27A	SCA27B
Inheritance pattern	AD	AD
*FGF14* genetic variant type	Conventional variants (missense, nonsense, insertion, deletion)	(GAA)_n_ STR expansion
Mean age of onset	12 years	60 years
Most prominent feature	Tremor	Gait ataxia
Episodic symptoms	21%	13–46%
Episode duration	Minutes-days	Minutes-days
Episode triggers	Fever	Alcohol, physical exertion, caffeine
Gait ataxia	90%	95–100%
Limb ataxia	82%	74%
Gaze-evoked nystagmus	86%	55%
Down-beat nystagmus	Rare	42%
Dysarthria	91%	50%
Vestibular hypofunction	n/a	22–60%
Other features	Dyskinesia (56%) – most commonly orofacial Cognitive impairment (50%) Psychiatric features – depression, aggressive outbursts	Autonomic features Lower limb pyramidal signs
Neuroimaging—cerebellar atrophy	Minority (20%)	Common (60–97%)
Treatment	Acetazolamide: limited reports of positive response	4-AP: response in majority Acetazolamide: response in some (44%)

AD: autosomal dominant; n/a: not available/unknown; 4-AP: 4-aminopyridine

Given the phenotypic differences in SCA27A and SCA27B, this suggests distinctions in their mechanisms of disease. Whilst SCA27A has been attributed to loss-of-function variants and haploinsufficiency, the mechanisms in SCA27B remain to be fully delineated [42, 61, 62]. In many other STR expansion disorders, such as the polyQ disorders, misfolding and aggregation of the abnormal proteins and RNA play a role in disease [19]. However, in SCA27B, post-mortem samples did not show intranuclear or cytoplasmic inclusions, but reduced *FGF14* RNA and protein expression, suggesting inhibition of transcription and primarily a loss-of function [8, 42]. Therefore, although both disorders are attributed to loss-of-function mechanisms, distinctions in pathologic and pathophysiologic processes, with consequent impacts on phenotype, remain to be determined. It is also of interest to draw a parallel with *CACNA1A*, which can result in the allelic disorders of SCA6, due to heterozygous (CAG)_n_ STR expansions, EA2, due to loss-of-function conventional variants and FHM, due to gain of function point mutations [63]. SCA6 and EA2 have overlapping phenotypes, and both can present with episodic ataxia, attributed to the common mechanism of impaired calcium channel function [63, 64]. However, it has been demonstrated that individuals with SCA6 pathologically demonstrate formation of aggregates, with intranuclear inclusions, and impairment in both channel regulation as well as altered transcriptional regulation of other genes [65].

### THAP11

A (CAG)_n_ STR expansion in exon 1 of the *THAP11* gene is a recently discovered AD HCA, and falls within the category of polyQ disorders [9, 38]. However, only a limited number of cases have been reported to date, including 24 affected individuals from two Chinese families [9]. A further case with ataxia and Parkinson’s disease was identified through the UK Biobank, but in addition to the *THAP11* STR expansion, they were also found to have a CAG STR expansion in *CACNA1A*, consistent with SCA6 [66]. Therefore, the significance of the *THAP11* STR expansion is uncertain in this individual. Based on reported cases to date, age at onset has ranged from 4–51 years, with a median age at onset of 34 years within the larger reported family, consisting of 22 family members [9, 66]. Clinical features include progressive gait and limb ataxia, dysarthria, with nystagmus reported in 33% (2/6) [9]. One individual had a resting bilateral upper limb tremor [9, 66]. In the larger reported family, one individual manifested a much more severe disease course, with onset at 4 years of age, and additional features of ptosis, slow saccades, dysphagia, pyramidal signs, myoclonic seizures, and cognitive impairment [9]. Cerebellar atrophy has been identified in all tested cases to date, whilst nerve conduction studies are generally normal [9]. The median age of death for deceased affected family members was 40 years (range 15–61), although the cause was not indicated [9].

STR expansion lengths of ≥ 45 repeats are considered pathogenic, whilst 17–39 repeats have been seen in healthy controls [9, 38, 66]. The pathogenicity of 40–44 repeats remains undetermined [9, 66]. Both affected and healthy individuals demonstrate CAA repeat interruptions, although the significance of these interruptions, and any disease-modulating effects remain unclear [9, 66].

In summary, (CAG)_n_ STR expansions in *THAP11* have recently been identified as a cause of SCA. However, only a limited number of patients have been reported, and further published cases will be required to define the full phenotypic spectrum and determine prevalence, including within broader ethnic and geographic groups [9, 38, 66].

### SCA4

**Fig. 1:**
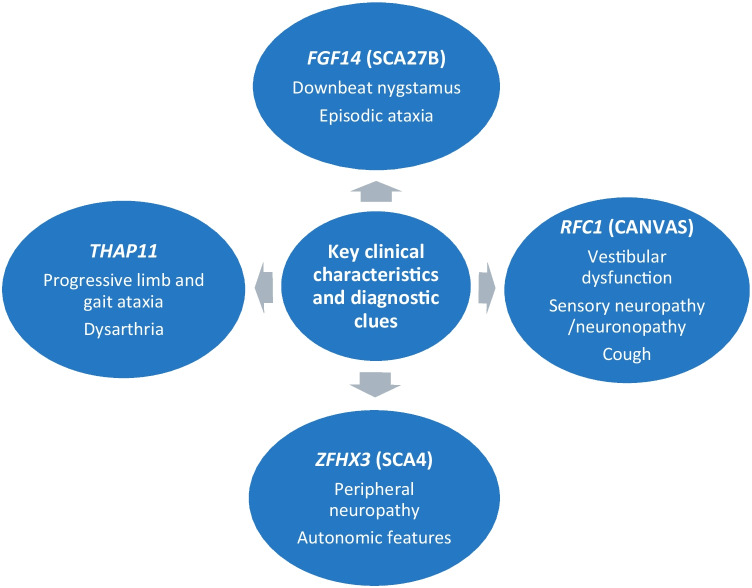
Recently described short tandem repeat expansions causing cerebellar ataxia with key diagnostic clues

Clinical descriptions of SCA4 were first published more than 25 years ago, but it is only recently, that a STR expansion in the last exon of *ZFHX3* was identified as the genetic basis for this disorder [10, 67, 68]. SCA4 presents with core features of gait and limb ataxia, and an axonal sensory neuropathy with hypo/areflexia [10, 69] (Fig. 1). Dysarthria and autonomic features are present in the majority, with upgoing-plantar responses present in some individuals, and nystagmus usually absent [10, 67, 69]. Individuals will commonly progress to wheelchair dependency [69]. Most reported individuals are of Swedish ancestry, attributed to the presence of a founder mutation, although other cases of non-Swedish European ancestry have been described [10, 69]. The cause for SCA4 has been identified as an exonic (GGC)_n_ STR expansion in *ZFHX3, *with ≥ 48 repeats considered pathogenic [10]. The normal range is identified as ≤ 31 repeats, whilst the pathogenicity of 32–47 repeats is presently unknown [10].

SCA4 appears to be a rare form of SCA, and has thus far, has only been identified in individuals with European ancestry. Testing of broader populations will be necessary for the determination of global prevalence.

### Autosomal Recessive Cerebellar Ataxias

The nomenclature for AR HCAs has been less consistent compared with the AD SCAs. Disorders have been labelled as sequentially numbered ‘autosomal recessive spinocerebellar ataxias’ (SCARs, OMIM series PS213200) or ‘autosomal recessive cerebellar ataxias’ (ARCAs). To clarify the nomenclature, a gene-based naming system has been proposed, utilising the gene name, preceded by a prefix describing the phenotype (e.g. ATX-*SYNE1*) [70]. However, there are challenges in determining the boundaries of classification where ataxia is a presenting feature, but there are broader or systemic features, including hereditary spastic paraplegia, neurodevelopmental disorders, metabolic and mitochondrial disorders, and encephalopathies, amongst others [71, 72]. Nevertheless, a 2019 consensus paper identified 59 primary ARCAs, and a further 48 complex disorders with multisystem involvement in which ataxia is a feature, and therefore should be considered in the evaluation [71]. Importantly, CANVAS, a relatively common AR ataxia due to STR expansions in *RFC1*, has been subsequently added to the list, and will be discussed in detail in the next section. AR-HCAs with potential adult onset have been included in Table 3.

**Table 3:** Adult-onset autosomal recessive hereditary cerebellar ataxias

Condition	Other names	Gene	Locus	Phenotype MIM
ATX-*ABHB12*	Polyneuropathy, hearing loss, ataxia, retinitis pigmentosa, and cataract (PHARC)	*ABHD12*	20p11.21	612674
ATX-*ADCK3*	ARCA2; SCAR9; Primary coenzyme Q10 deficiency-4 (COQ10D4)	*ADCK3*	1q42.13	612016
ATX-*ANO10*	ARCA3; SCAR10	*ANO10*	3p22.1–21.33	613728
ATX-*APTX*	AOA1	*APTX*	9p21.1	208920
ATX-*ATM*	Ataxia-telangiectasia (AT)	*ATM*	11q22.3	208900
ATX-*C10orf2*	Mitochondrial DNA depletion syndrome-7 (MTDPS7)	*TWNK*	10q24.31	271245
ATX-*CWF19L1*	SCAR17	*CWF19L1*	10q24.31	616127
ATX-*CYP27A1*	Cerebrotendinous xanthomatosis (CTX)	*CYP27A1*	2q35	213700
ATX-*FXN*	Friedreich ataxia (FRDA)	*FXN*	9q21.11	229300
ATX-*GDAP2*	SCAR27	*GDAP2*	1p12	618369
ATX-*GRID2*	SCAR18	*GRID2*	4q22.1–22.2	616204
ATX-*GRN*	Neuronal ceroid lipofuscinosis-11 (CLN11)	*GRN*	17q21.31	614706
ATX-*L2HGDH*	L-2-hydroxyglutaric aciduria (L2HGA)	*L2HGDH*	14q21.3	236792
ATX-*MAN2B1*	Alpha-mannosidosis	*MAN2B1*	19p13.13	248500
ATX-*NPC1*	Niemann-Pick disease type C1 (NPC1)	*NPC1*	18q11.2	257220
ATX-*NPC2*	Niemann-Pick disease type C2 (NPC2)	*NPC2*	14q24.3	607625
ATX-*PEX7*	Refsum disease	*PEX7*	6q23.3	614879
ATX-*PHYH*	Refsum disease	*PHYH*	10p13	266500
ATX-*PIK3R5*	AOA3	*PIK3R5*	17p13.1	615217
ATX-*RFC1*	Cerebellar ataxia, neuropathy and vestibular areflexia syndrome (CANVAS)	*RFC1*	4p14	614575
ATX-*RNF216*	Cerebellar ataxia and hypogonadotrophic hypogonadism; Gordon Holmes syndrome	*RNF216*	7p22.1	212840
ATX-*SETX*	AOA2; SCAR1; SCAN2	*SETX*	9q34.13	606002
ATX-*STUB1*	SCAR16	*STUB1*	16p13.3	615768
ATX-*SYNE1*	ARCA1; SCAR8; recessive ataxia of Beauce	*SYNE1*	6q25.2	610743
ATX-*TTPA*	Ataxia with vitamin E deficiency (AVED)	*TTPA*	8q12.3	277460
ATX-*TTC19*	Mitochondrial complex III deficiency nuclear type 2 (MC3DN2)	*TTC19*	17p12	615157
ATX-*XRCC1*	SCAR26	*XRCC1*	19q13.31	617633
ATX/HSP-*DARS2*	Leukoencephalopathy with brain stem and spinal cord involvement and lactate elevation (LBSL)	*DARS2*	1q25.1	611105
ATX/HSP-*HEXA*	Tay-Sachs disease	*HEXA*	15q23	272800
ATX/HSP-*HEXB*	Sandhoff disease	*HEXB*	5q13.3	268800
ATX/HSP-*PNPLA6*	Boucher-Neuhauser syndrome	*PNPLA6*	19p13.2	215470
ATX/HSP*-SACS*	Autosomal recessive spastic ataxia of Charlevoix-Saguenay (ARSACS); SPAX6	*SACS*	13q12.12	270550
ATX/HSP-*VPS13D*	SCAR4	*VPS13D*	1p36.22–36.21	607317
HSP/ATX-*CAPN1*	SPG76	*CAPN1*	11q13.1	616907
HSP/ATX-*CLCN2*	Leukoencephalopathy with ataxia (LKPAT)	*CLCN2*	3q27.1	615651
HSP/ATX*-CYP7B1*	SPG5	*CYP7B1*	8q12.3	270800
HSP/ATX-*KIF1C*	SPAX2	*KIF1C*	17p13.2	611302
HSP/ATX-SPG7	SPG7	*PGN*	16q24.3	607259
ATX/MYC-*TPP1*	SCAR7	*TPP1*	11p15.4	609270
MYC/ATX-*NEU1*	Sialidosis type I	*NEU1*	6p21.33	256550
DYT/ATX*-ATP7B*	Wilson disease	*ATP7B*	13q14.3	277900
*MTTP*	Abetalipoproteinemia	*MTTP*	4q23	200100
*POLG*	Mitochondrial recessive ataxia syndrome (MIRAS); sensory ataxic neuropathy, dysarthria and ophthalmoparesis (SANDO); spinocerebellar ataxia with epilepsy (SCAE)	*POLG*	15q26.1	607459
SPAX2	Autosomal recessive spastic ataxia with leukoencephalopathy (ARSAL)	*MARS2*	2q33.1	611390
SPAX10	-	*COQ4*	9q34.11	620666

AOA: ataxia-oculomotor apraxia; ARCA: autosomal recessive cerebellar ataxia; SCAR: autosomal recessive spinocerebellar ataxia; SCAN: spinocerebellar ataxia with axonal neuropathy; SPAX: spastic ataxia; SPG: spastic paraplegia

Onset of the AR HCAs usually occurs before 40 years of age and cases are often sporadic, without family history [73]. In addition to the core feature of cerebellar ataxia, the AR HCAs demonstrate greater phenotypic variability than the AD HCAs, including other neurologic and non-neurologic features [74]. The majority of AR HCAs are due to conventional variants (i.e. non-STR expansions) [74]. However, both CANVAS and Friedreich ataxia (FRDA) are caused by biallelic STR expansions in *RFC1* and *FXN* respectively, and reflect two of the most common causes of AR HCAs. Several genes have now also been reported to cause both AD and AR ataxias, including *AFG3L2*, *SPTBN2* and *ITPR1* [73]. Generally, the AR forms of these disorders are more severe, with earlier onset and additional clinical features, but this highlights the potential complexity in determining the significance of heterozygous variants in such genes [73].

## *RFC1-*Related Cerebellar Ataxia, Neuropathy, Vestibular Areflexia Syndrome (CANVAS)

Biallelic STR expansions in intron 2 of *RFC1* have recently been identified as a relatively common cause of adult-onset ataxia, causing CANVAS, named after the three core features of this disorder [7, 11]. In the original report, *RFC1-*CANVAS was genetically confirmed in 22% of individuals with sporadic late-onset ataxia [7]. Subsequent studies have reported more variable prevalence (1–29%), which appears to depend on testing criteria, with increased yield seen in those with cerebellar ataxia in addition to at least one of bilateral vestibulopathy or peripheral neuropathy, and highest yields in those with all three core features (73–100%) [75].

Onset of neurologic features occurs at a mean of 52–54 years (range 19–76) [7, 30, 76]. Individuals may present with 1–3 of the core features, with combinations of cerebellar, sensory, and vestibular ataxia contributing to impaired gait and coordination. Sensory impairment can be profound, with neurophysiologic testing often demonstrating generally absent sensory action potentials, whilst bilateral vestibular areflexia is seen in 54–88% [7, 30]. Cough is a common feature (64–72%) and can pre-date ataxia by decades, with mean onset at 35 years [30, 76]. Dysarthria occurs in 40% and oscillopsia in 33% [30]. Other features include autonomic involvement (23–62%) and extrapyramidal features, including bradykinesia (27%) [7, 30, 76]. Cerebral imaging shows cerebellar atrophy in 83% [7]. Progress is slow, with a half of patients requiring a stick at 10 years after onset, and a quarter requiring a wheelchair at the 15-year mark [30].

CANVAS is typically caused by a biallelic intronic (AAGGG)_n_ STR expansion in *RFC1* [7]. A repeat length of ≥ 400 repeats has been identified as pathogenic, although the lower limit of pathogenicity remains to be delineated [7]. The (AAGGG)_n_ STR expansion has a carrier frequency of 0.7–6.5% [7, 75]. Other less common pathogenic motifs have been identified, as well as motifs that are benign and of undetermined pathogenicity state (Table 4) [7, 77–81]. Furthermore, recent reports have identified individuals with a CANVAS phenotype, who were heterozygous for the (AAGGG)_n_ STR expansion and had a truncating variant in the second allele [82, 83].

**Table 4:** Pathogenicity classification of *RFC1* short tandem repeat expansion motifs

Classification	Repeat motif	Ancestry/ethnicity for pathogenic variants
Pathogenic	(AAGGG)_≥400_	Various populations/common
	(ACAGG)_n_	Asian-Pacific, Japanese
	(AGGGC)_n_	Caucasian, mixed
	(AAGGC)_n_	South Asian
	(AGAGG)_n_	Caucasian
	(AAAGG)_10–15_(AAGGG)_n_(AAAGG)_4–6_	New Zealand and Cook Island Māori
	(AAAGG)_≥600_	Caucasian
Benign	Smaller (AAAGG)_n_^*^	
	(AAAAG)_n_	
	(AAGAG)_n_	
	(AAAGGG)_n_	
Uncertain pathogenicity	(AGAAG)_n_	
	(AACGG)_n_	

^*^Threshold not defined

Biallelic (ACAGG)_n_ STR expansions were identified in three symptomatic family members, who presented with a CANVAS-plus phenotype; showing additional features of fasciculations and elevated levels of creatine kinase [78]. This raises the possibility that less common motifs may be associated with atypical clinical features. However, in another case also found to have biallelic (ACAGG)_n_ STR expansions, atypical features were not reported, so further case reports for this motif will assist genotype–phenotype correlation [84]. Despite the expanding knowledge of underlying pathogenic *RFC1* variants, the disease mechanisms remain unknown. Furthermore, clinical variability with different pathogenic motifs, and interactions between alleles harbouring a STR expansion, and alleles with a conventional variant, remain to be determined [75].

### Genetic Testing Approach

Before considering genetic causes of ataxia, and proceeding to genetic testing, acquired and non-genetic causes must be excluded. The diagnostic approach to ataxias as a general group, and the evaluation of these acquired causes, is not discussed here, but has been described elsewhere [85, 86]. When the decision is made to proceed to genetic testing, the first important steps are to determine whether there is a family history of ataxia, and the pattern of inheritance, or whether the individual is a sporadic case, as this will determine the appropriate genes to test. We acknowledge that testing approaches may also be limited by the availability of testing methods and local experience, and the recommendations that follow may require adjustment in circumstances where specific testing is unavailable.

During clinical evaluation, certain clinical features or biomarkers may be identified, which lead to high suspicion for a particular genetic cause (Table 5). In such cases, it may be more resource-efficient to test for the single or several genes that are suspected, and if negative, then proceed to a broader testing pathway. It is generally recommended that all patients undertake blood tests, for exclusion of acquired causes as well as evaluation of biochemical markers for genetic ataxias. For example, low vitamin E levels are suggestive of ATX-*TTPA* (ataxia with vitamin E deficiency, AVED) and abetalipoproteinemia (*MTTP*), whilst elevated alpha-fetoprotein (AFP) is seen in ATX-*ATM* (ataxia telangiectasia, AT), with milder/variable elevations also seen in ATX-*APTX* (ataxia-oculomotor apraxia type 1, AOA1), ATX-*SETX* (AOA2), ATX-*PIK3R5* (AOA3), and rarely in ATX-*ANO10* (ARCA3)[87–90]. It is also recommended all individuals complete a brain MRI to exclude acquired causes, but results from MRI can also suggest certain genetic diagnoses. For example, T2 hyperintensities of bilateral middle cerebellar peduncles may suggest FXTAS, whilst T2-hypointensite stripes in the pons are suggestive of ATX/HSP-*SACS* (AR spastic ataxia of Charlevoix-Saguenay, ARSACS) [73, 91]. The presence of multiple T2 hypointense punctate foci visible on SWI, indicative of cerebral microhaemorrhages and haemosiderin deposition, also points to ATX-*ATM* (AT) [92, 93]. Although clinical evaluation may identify highly suggestive features for certain individuals, many cases remain undefined, and a broader testing approach is recommended, as next described.

**Table 5:** Biomarkers suggestive of specific genetic ataxias

Biomarker	Result	Phenotype/Gene	References
Blood tests			
Albumin	↓	AOA1 (*APTX*)*,* AOA2 (*SETX*)	[88–90]
AFP	↑	AOA1 (*APTX*)*,* AOA2 (*SETX*)*,* AOA3 (*PIK3R5*), AOA4 (*PNKP*), ARCA3 (*ANO10*), AT (*ATM*)	[88–90]
Ceruloplasmin	↓	Wilson disease (*ATP7B*)	[73, 90]
Cholestanol	↑	Cerebrotendinous xanthomatosis (*CYP27A1*)	[90]
Cholesterol	↓ ↑	Abetalipoproteinemia (*MTTP*) AOA1 (*APTX*)*,* AOA2 (*SETX*)	[88–90]
Creatine kinase	↑	AOA2 (*SETX*), ARCA2 (*ADCK3*)	[89, 90, 112]
Copper	↓	Wilson disease (*ATP7B*)	[90]
Ferritin	↑	Aceruloplasminemia (*CP*)	[113]
Immunoglobulins	↓	AT (*ATM*)	[89]
Iron	↓	Aceruloplasminemia (*CP*)	[90]
Lactate	↑	ARCA2 (*ADCK3*), LSBL (*DARS2*), *POLG*, *RRM2B, TWNK*, *TTC19*, mtDNA variants	[70, 90, 114–116]
Vitamin E	↓	AVED (*TTPA*), abetalipoproteinemia (*MTTP*)	[87, 90]
VLCFA	↑	Adrenoleukodystrophy (*ABCD1*)	[117]
Urine tests			
Copper	↑	Wilson disease (*ATP7B*)	[90]
MRI brain	MCP T2 hyperintensities	FXTAS (*FMR1*)	[91]
	Pontine T2 linear hypointensities	ARSACS (*SACS*)	[73]
	Punctate foci of haemosiderin	AT (*ATM*)	[92, 93]
	Stroke-like lesions	*POLG*, mtDNA variants	[115]
	Inferior olivary nuclei hyperintensities	*POLG*	[115]

↑: increased; ↓: decreased; AFP: alpha-fetoprotein; AOA: ataxia-oculomotor apraxia; ARCA: autosomal recessive cerebellar ataxia; ARSACS: autosomal recessive spastic ataxia of Charlevoix-Saguenay; AT: ataxia-telangiectasia; AVED: ataxia with vitamin E deficiency; FXTAS: Fragile X tremor/ataxia syndrome; LSBL: leukoencephalopathy with brainstem and spinal cord involvement and lactate elevation; MCP: middle cerebellar peduncles; VLCFA: very long-chain fatty acids

**Fig. 2:**
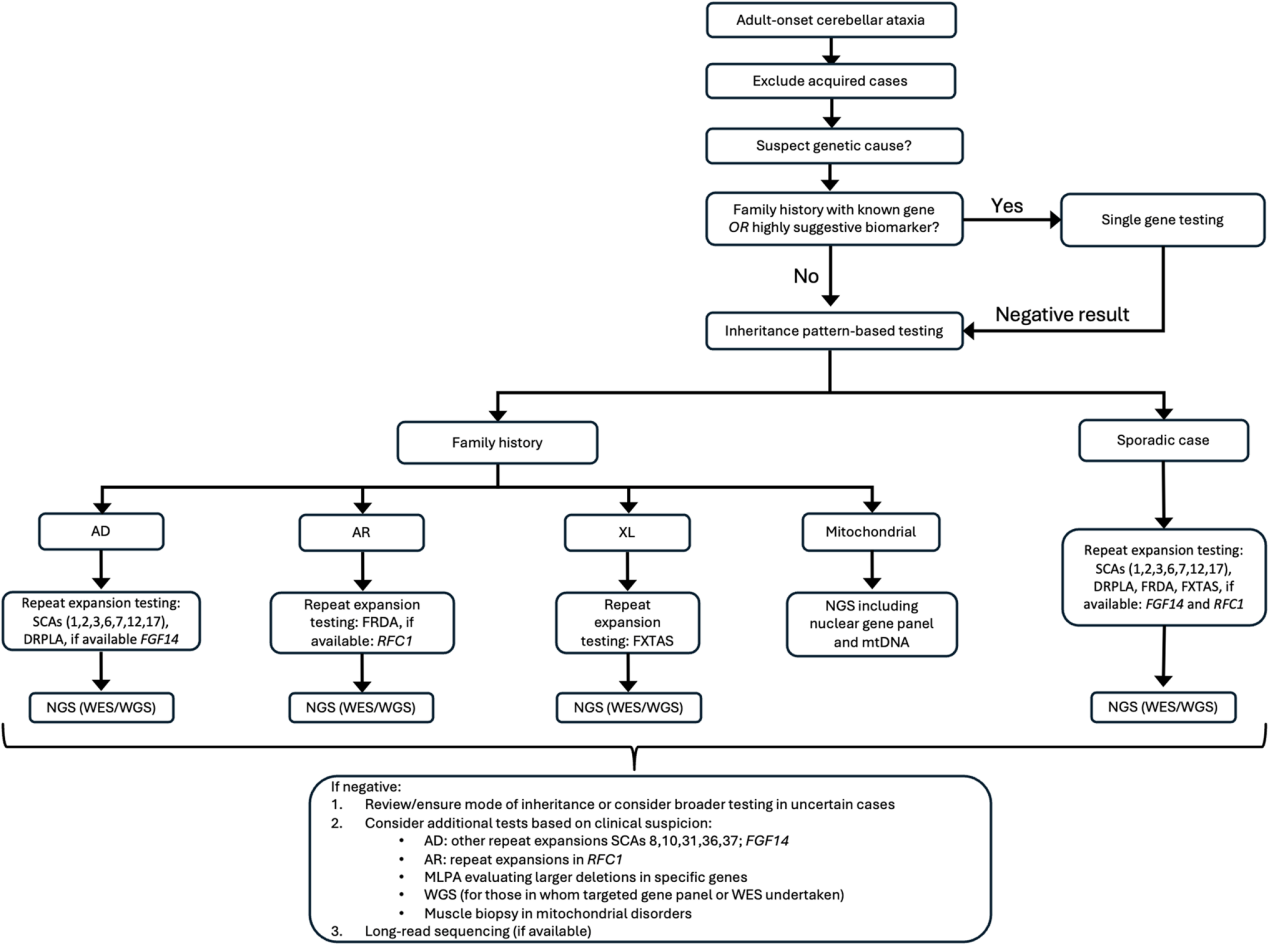
Flowchart of current suggested testing approach for genetic diagnosis of adult-onset hereditary cerebellar ataxia. **[**AD: autosomal dominant; AR: autosomal recessive; DRPLA: dentatorubral-pallidoluysian atrophy; FRDA: Friedreich ataxia; FXTAS: fragile X-associated tremor/ataxia syndrome; mtDNA: mitochondrial DNA; NGS: next-generation sequencing; SCA: spinocerebellar ataxia; WES: whole exome sequencing; WGS: whole genome sequencing; XL: X-linked]

For cases with a positive family history, in which a specific gene has been identified, single-gene testing can be undertaken. However, in families where the gene is not known/suspected and family history is suggestive of AD inheritance, it is recommended to commence with a panel for STR expansions in the more common SCAs [1–3, 6, 7, 10, 16] and DRPLA [12, 17]. In some laboratories, testing for other SCAs, including SCA8, 10, 31, 36 and 37 may also be available, although is often not included on routine panels [12, 17]. Furthermore, as *FGF14* STR expansions appear to be a relatively common cause of late-onset HCA, if testing is locally available, we would recommend testing of *FGF14* concurrent to other AD STR expansions (Fig. 2). In the case of AR inheritance, FRDA should be tested in the first instance, and we recommend testing for *RFC1* STR expansions if locally available, although acknowledge this is presently not the case in many centres, including in the authors’ own experience [12]. For individuals in whom X-linked inheritance is suspected, or subsequent generations present with intellectual disability or other features suggestive of Fragile X Syndrome, testing for STR expansions in *FMR1* (for FXTAS) should be considered. In sporadic cases, a combination of these tests is recommended, including the SCA STR expansion panel, DRPLA, FRDA and FXTAS, as well as *FGF14* and *RFC1*, if testing is accessible [12].

If a causative STR expansion is not identified, individuals should proceed to next generation sequencing [12, 17, 85]. The modality of testing will likely depend on local availability and expertise but would most commonly involve either testing a targeted panel of ataxia genes, or whole exome sequencing. In some centres, whole genome sequencing may be undertaken, although due to higher cost, this is less commonly utilised in current standard clinical practice [17].

For cases remaining undiagnosed, testing for specific additional genes may be considered but will be dependent on consistency of phenotype and mode of inheritance, as well as availability. This includes testing additional STR expansions for SCA8, 10, 31, 36 and 37, *FGF14* and *RFC1* if not already tested during initial evaluation. Furthermore, knowledge of the geographic origin of the patient may also guide testing decisions, given certain regions are associated with much higher relative frequency of HCA genetic subtypes. For example, SCA4 has most commonly been described in individuals of Swedish ancestry [68], SCA31 in individuals from Japan [94], SCA36 in patients from Japan or Spain [95], and SCA37 in those from Portugal or Spain [96]. For individuals with a single STR expansion identified in *RFC1* or *FXN*, and with a phenotype suggestive of CANVAS or FRDA respectively, it is recommended that individuals undergo further genetic testing for conventional variants in the other allele. In such cases, it may be beneficial and diagnostically efficient for laboratories to complete reflex sequencing (i.e. sequencing is undertaken automatically as a next step) for *RFC1* or *FXN*, respectively. MLPA may be considered to evaluate larger gene deletions, such as in *ITPR1* (for SCA15/16) [17]. In some cases, variants in mtDNA (e.g. *MT-TL1)* may not be detectable by blood testing and require a muscle biopsy for genetic diagnosis [97]. However, given the invasive nature of the procedure, for cases with a low suspicion (e.g. absence of other systemic features, family history inconsistent for maternal inheritance), this invasive procedure is not routinely recommended.

**Fig. 3:**
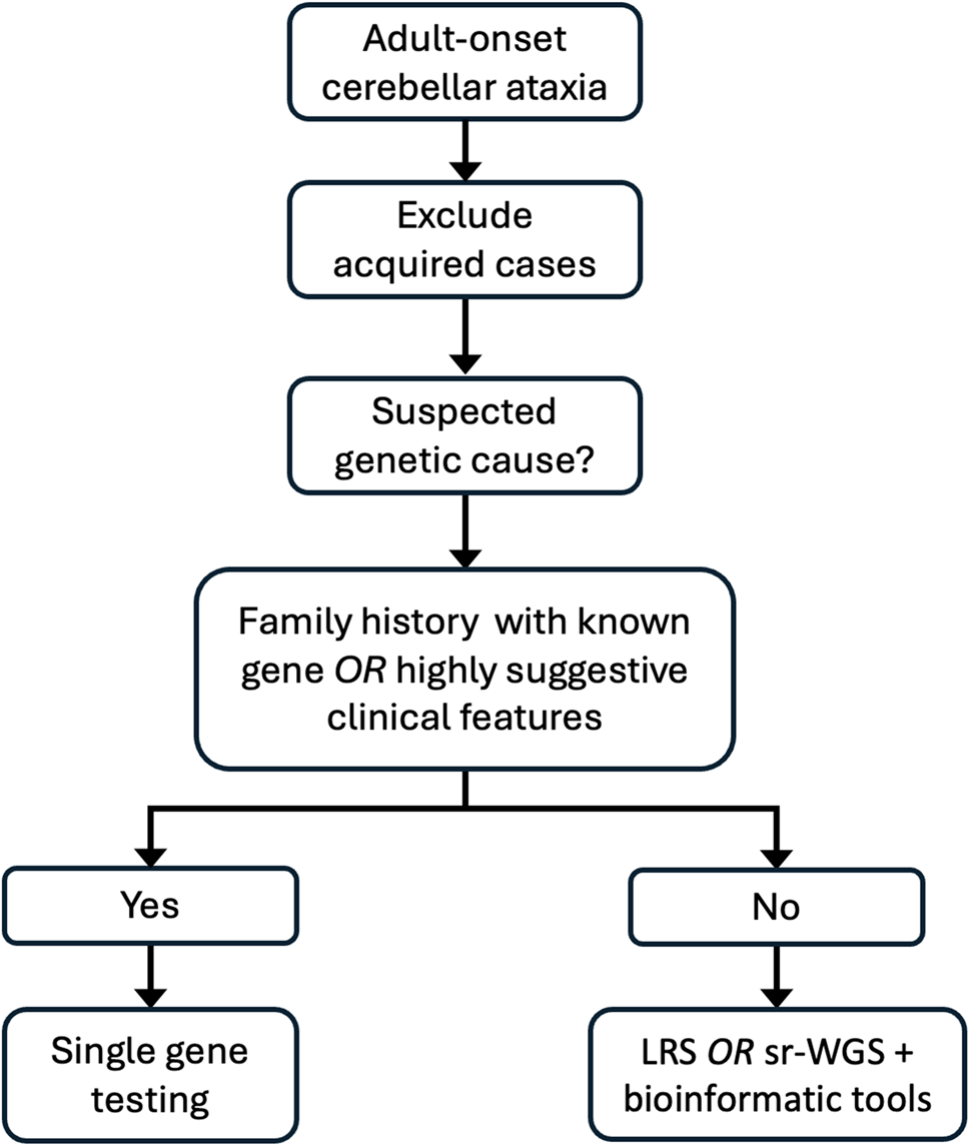
Potential future testing approach for genetic diagnosis of adult-onset hereditary cerebellar ataxia using advanced genomic technologies.** [**LRS: long-read sequencing; sr-WGS: short-read whole genome sequencing]

Despite undertaking this sequential multi-test approach, some individuals remain undiagnosed due to gene variants being missed on account of limitations of the genetic testing methods. For example, deep intronic variants are not adequately evaluated with whole exome sequencing, although would be detectable with whole genome sequencing [98]. Furthermore, larger deletions and structural variants may be more accurately characterised using long-read sequencing (LRS) in comparison to short-read sequencing [99]. LRS has been shown to have high sensitivity for the detection of STR expansions, with the ability to determine STR length, including for large expansions (e.g. *RFC1-*related CANVAS), as well as sequencing of the region, permitting identification of the repeat motif and presence of interruptions, which may be relevant for prediction of pathogenicity [100]. Furthermore, this dual capacity may be relevant for cases of FRDA and *RFC1-*related CANVAS, which may be caused by a monoallelic STR expansion in combination with a conventional variant on the other allele. Several different LRS testing approaches have demonstrated utility, including whole-genome LRS and ‘adaptive’ or ‘Read Until’ sampling [101, 102]. In adaptive sampling, regions of interest can be targeted using real-time software-based selection, permitting selective sequencing of targets without any need for laboratory-based target enrichment [100, 102]. An alternative method, includes the use of CRISPR-Cas9 for target enrichment within a sample, prior to LRS [96]. Both of these methods avoid the use of PCR-dependent target enrichment, which can be challenging in repetitive and/or GC-rich regions, such as STRs, and result in reduced coverage [99, 102]. This contributes to the improved uniformity of coverage with use of LRS compared to short-read next generation sequencing, which generally requires a PCR step [99]. Furthermore, without the need for PCR-based amplification, LRS can identify methylation patterns of native DNA [99]. LRS of complementary DNA to RNA from peripheral blood, has also been used in functional testing, to support the mechanism of nonsense-mediated decay [101]. There are, however, limitations with LRS, including a reduced read-level sequencing accuracy compared to next-generation sequencing (although we note that the gap has narrowed rapidly in recent years), and access to LRS technologies is currently relatively limited [102, 103]. Presently, there are two leading long-read sequencing platforms: Pacific Biosciences and Oxford Nanopore Technologies, which have both demonstrated utility in genomic diagnostics [99]. We expect these technologies will continue to develop, but the optimal platform remains to be determined. Long-read sequencing appears to be a particularly promising technology in the evaluation of ataxias, given the ability to detect almost all variant types including STR expansions, conventional variants, and structural variants, within a single test, as well as methylation profiles. It is possible that long-read sequencing may supersede the sequential testing approach altogether, with individuals undergoing long-read sequencing as the initial test, in all cases where a single gene is not highly suspected (Fig. 3).

Alternatively, the development of advanced bioinformatics tools (e.g. ExpansionHunter, GangSTR, HipSTR, amongst others) has allowed improved detection of STR expansions using short-read next generation sequencing (srNGS), including both whole genome sequencing and whole exome sequencing [104, 105]. SrNGS combined with advanced bioinformatics tools may therefore provide an alternative first-line test for individuals presenting with adult-onset HCA (Fig. 3). However, limitations remain, in that for larger STR expansions, the accuracy of repeat length estimation is reduced, with a tendency to underestimate repeat length [104]. Whilst long-read sequencing and short-read whole genome sequencing both offer potential advantages in the evaluation of ataxias and will likely alter testing approaches in future practice, presently, use of these technologies is constrained by availability and cost. The above suggested clinical testing approach, incorporating whole exome sequencing, provides a clinically available compromise for cost and yield [20].

### Diagnostic Challenges and Future Directions

**Fig. 4:**
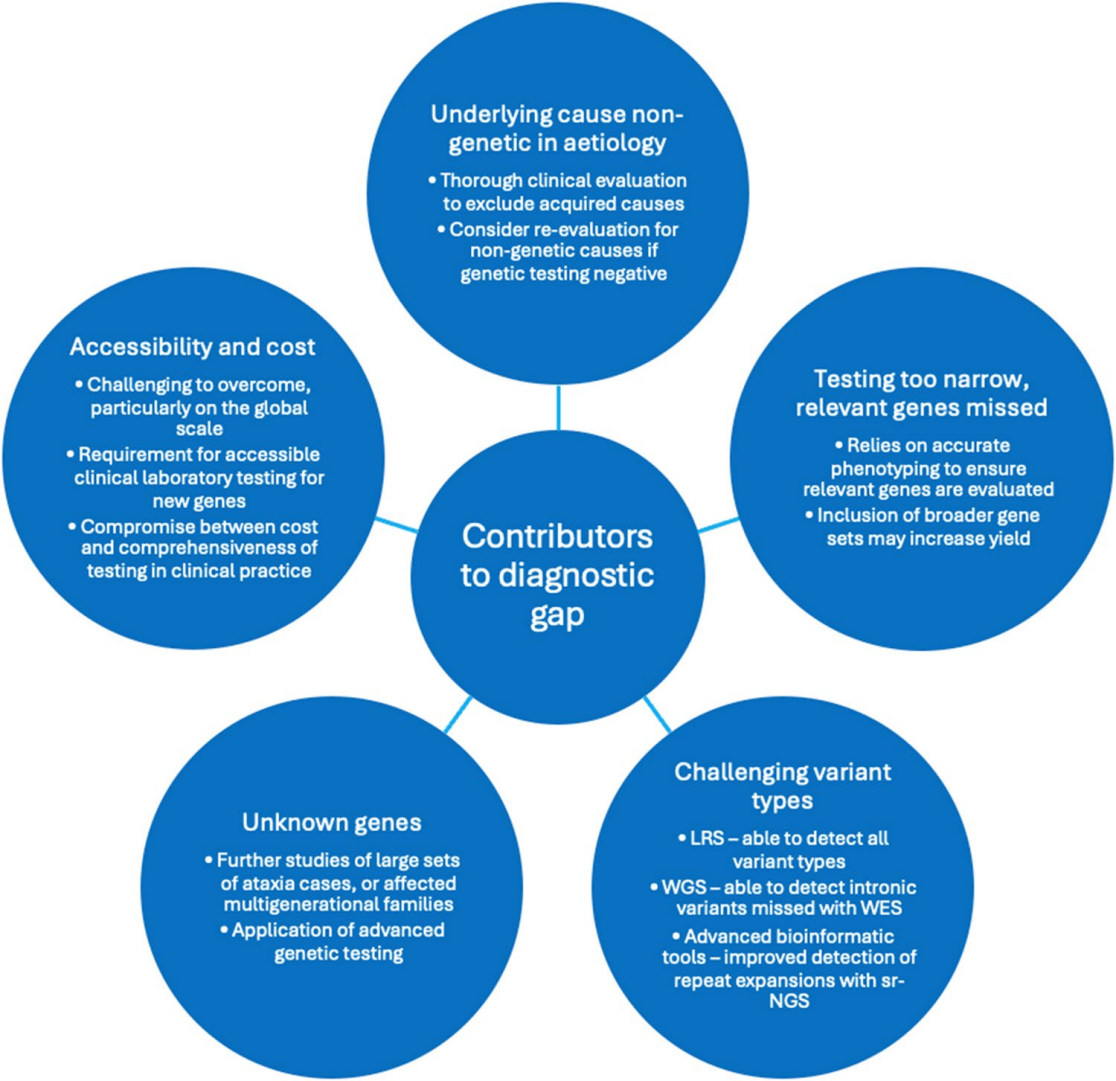
Contributing factors and potential solutions for the diagnostic gap in hereditary cerebellar ataxias.** [**HSP: hereditary spastic paraplegia; LRS: long-read sequencing; sr-NGS: short-read next-generation sequencing; STR: short tandem repeat; WES: whole exome sequencing; WGS: whole genome sequencing]

Following genetic testing, 29–71% of individuals with a suspected HCA remain without a genetic diagnosis [12–15]. Furthermore, the yield is typically higher in individuals with young onset and family history, and lower in late-onset and sporadic cases [13]. A genetic diagnosis permits individuals to receive more accurate disease-specific prognostic information, has potential implications for treatments and monitoring recommendations, affects eligibility for gene-specific treatment trials, and has implications for family risk counselling and reproductive considerations [106]. It is therefore important to continue narrowing this diagnostic gap. In the earlier section of this review, we described the potential for missed diagnoses related to limitations of genetic testing modalities, but here we describe the additional factors that may contribute to the diagnostic gap, and potential solutions (Fig. 4).

A thorough and accurate clinical evaluation is important to ensure non-genetic or acquired aetiologies are appropriately excluded, but also to correctly identify the presenting phenotype, which determines which genes are to be evaluated by genetic testing. Several genes are also associated with a broad spectrum of clinical features, and case reports of atypical presentations continue to expand the known clinical spectrum of various genetic disorders. For example, *SPG7* was initially associated with hereditary spastic paraplegia, but 15 years later was also found to cause cerebellar ataxia, whilst FXTAS, has also been reported to cause features of spastic paraparesis [107, 108]. Given that ataxias and hereditary spastic paraplegia have overlapping features, testing with a broader spastic-ataxia spectrum disorder gene set may identify the genetic cause in those with atypical or less common phenotypic features. We must also acknowledge that availability of testing may limit our ability to test for known gene variants in routine practice. From the authors’ experience, commercial testing is not currently available within Australia for *RFC1* and *FGF14* STR expansions, both relatively common causes of late-onset HCA. Therefore, from a practical perspective, availability and financial constraints pose a real impact on the ability to achieve higher diagnostic rates within clinical practice.

Finally, numerous causative genes or gene variants may remain undetected. Further studies evaluating large sets of undiagnosed ataxia cases, as well as multigenerational genetic evaluation in affected families may uncover further genetic causes. Advances in genetic testing technologies have led to the discovery of several new ataxia genes in recent years, and this is particularly well exemplified in SCA4. Although the disease locus was identified more than 25 years ago, it is only with newer testing technologies including long-read sequencing and short-read whole genome sequencing combined with advanced bioinformatics tools, that the causative gene and gene variant were finally discovered [10,, 68]. Given the improved capabilities of these advanced genetic technologies to detect variants classically considered more challenging to detect, including STR expansions, intronic variants, complex structural variants, or even potentially relevant epigenetic changes, we anticipate that ongoing gene discovery in the field of HCAs will continue to narrow the diagnostic gap.

## Conclusion

The process of genetic evaluation for individuals with adult-onset HCA is often complex. There is a large number potential causative genes, which includes a rapidly expanding list of repeat expansion disorders (e.g. SCA27B), posing distinct challenges for a genetic diagnosis. These challenges could be addressed by developments in genetic testing methods, including improvements in bioinformatic tools for short-read sequencing analysis and the emergence of long-read sequencing. The generation of one-stop comprehensive genetic tests, or tests that are readily adaptable to the discovery of new repeat expansion disorders, will be key to improving the diagnostic yield in HCA.

## Data Availability

Data sharing is not applicable as no new data were generated during this study.
